# Serum selenium level and cancer risk: a nested case-control study

**DOI:** 10.1186/s13053-019-0131-7

**Published:** 2019-12-23

**Authors:** Steven A. Narod, Tomasz Huzarski, Anna Jakubowska, Jacek Gronwald, Cezary Cybulski, Oleg Oszurek, Tadeusz Dębniak, Katarzyna Jaworska-Bieniek, Marcin Lener, Katarzyna Białkowska, Grzegorz Sukiennicki, Magdalena Muszyńska, Wojciech Marciniak, Ping Sun, Joanne Kotsopoulos, Jan Lubiński

**Affiliations:** 1 Women’s College Research Institute, Toronto, Canada; 2 Dalla Lana School of Public Health, University of Toronto, Toronto, Canada; 3 Department of Genetics and Pathology, International Hereditary Cancer Center, Pomeranian Medical University, Szczecin, Poland; 4 Read-Gene SA, Grzepnica, Szczecin, Poland; 5 Independent laboratory of Molecular Biology and Genetic Diagnostics, Pomeranian Medical University, Szczecin, Poland

**Keywords:** Selenium, *BRCA1*, Cancer, Poland

## Abstract

**Background:**

Epidemiologic studies have demonstrated a relationship between selenium status and cancer risk among those with low selenium levels. It is of interest to prospectively evaluate the relationship between selenium and cancer among women who reside in a region with ubiquitously low selenium levels.

**Methods:**

We performed a nested case-control study of baseline serum selenium levels and cancer risk using data and biological samples from 19,573 females that were participants in a biobanking initiative between 2010 and 2014 in Szczecin Poland. Cases included women with any incident cancer (*n* = 97) and controls (*n* = 184) were women with no cancer at baseline or follow-up. Serum selenium was quantified using mass spectroscopy.

**Results:**

The odds ratio associated being below the cutoff of 70.0 μg/L compared to a level above 70.0 μg/L was 2.29 (95% CI 1.26–4.19; *P* = 0.007). The risks for women in the two middle categories were similar and suggests that the normal range be between 70 μg/L and 90 μg/L. There was evidence for an increased risk of cancer among women in the highest category of selenium levels (i.e., > 90 μg/L), but this association did not achieve statistical significance (OR = 1.63; 95%CI 0.63–4.19; *P* = 0.31).

**Conclusions:**

Results from this study suggest that suggest that the optimum serum level of selenium in women living in Poland should be between 70 μg/L and 90 μg/L.

## Introduction

Selenium is an essential trace element with numerous health-related functions [1]. It has been proposed that selenium has cancer preventive properties, due the role of this nutrient as a cofactor of several antioxidant enzymes [1, 2]. Although the epidemiological evidence is inconclusive with respect to the health benefits of selenium, there is continued interest in exploring the chemo-protective properties of selenium for specific cancers including cancers of the prostate, lung, pancreas, liver, skin, head and neck, bladder, colon and breast [2–10]. When evaluating the relationship between selenium and cancer incidence (or mortality or prognosis) various factors can impact upon the findings, including the method of assessment of selenium status (i.e., dietary vs. blood selenium) as well as the geographic location and baseline characteristics of the study population [2–11]. Blood selenium represents a valid biomarker of selenium status because it reflects the selenium content of food, which in turn is determined by the selenium content of the soil and this has been shown to differ dramatically between locations [11, 12]. Many of the studies to date which demonstrate no cancer protective effect (or a potentially adverse effects of a high selenium level) have been conducted in regions where selenium levels in the soil are relatively high and/or in countries with high rates of supplement use [4, 13].

We have previously reported low serum selenium levels in patients with colon, lung and laryngeal cancer in Estonia and Poland, two regions of Europe known to have inherently lower selenium levels compared to North American countries [1, 14, 15]. Although these findings provide provocative preliminary evidence to support a potential beneficial effect of selenium supplementation in individuals with low selenium status residing in regions with low selenium levels, one limitation of these case-control studies was the retrospective nature of the analyses, whereby the assessment of selenium status in the blood followed the diagnosis of cancer. It is of interest to establish, in a prospective study, if selenium status prior to a cancer diagnosis has an effect on cancer risk in women in a country with ubiquitously low selenium levels. The goal of the current study was to evaluate the relationship between serum selenium levels and subsequent cancer risk in a large cohort of women followed for incident cases of cancer in Szczecin Poland, using a nested case-control study design.

## Patients and methods

### Study population

A large biobanking initiative began in 2010 as an adjunct study to complement the clinical records maintained on patients seen at the Cancer Genetic Outpatient Clinic of the University Hospital of the Pomeranian Medical University in Szczecin, Poland. Each patient who attended the outpatient clinic was invited to participate. Patients who attended the clinic were referred by their family doctor or a specialist for the evaluation of cancer risk and for genetic testing, including women at all levels of risk. The vast majority of patients who attended the clinic underwent genetic testing for three Polish *BRCA1* founder mutations (c.5266dupC, c.4035delA, c.181T>G); as part of the testing protocol, the patients were asked permission to store the residual serum to be included in the biobank. Each subject provided written consent for biobanking for research purposes. Patients were asked to fast for four hours prior to blood collection (10 cc). Blood samples were taken between 8 am and 2 pm and was centrifuged within 30 to 120 min to separate the serum from the cellular fraction. The serum samples were stored at -80^o^ until the selenium assay was conducted. For most patients, blood was taken once, but for some, samples were taken on multiple occasions corresponding to different visits. In the present study, if more than one sample was available, we used the first sample for the selenium assay. A total of 33,062 participated in the biobank of whom 19,573 were women with no previous cancer diagnosis at the time of blood collection.

All patients were followed for new cancers through a systematic review of pathology records for incident cancers at the three major cancer hospitals in the Szczecin region. Subjects in the biobank were linked to the records of the pathology department using a unique eleven digit identification number (PESEL). Women at high risk for breast cancer were eligible to be enrolled in a screening protocol which included annual mammography, breast ultrasound, CA125 and trans-vaginal ultrasound. MRI screening was restricted to women with a *BRCA1* mutation.

### Case and control subjects

Study subjects for the present nested case-control study were women at risk for breast cancer who attended the Cancer Genetics Outpatient clinic between 2010 and 2014. Cases were women with any incident cancer identified from among the women enrolled in the biobank who had no history of cancer at the time of the blood draw. For each case, we selected two controls from among those women with no cancer at baseline or at follow-up. Cases and controls were matched on year of birth (within two years), smoking status (current or past, pack years) and family history of cancer (number of affected first-degree relatives).

### Selenium assay

Serum selenium levels were measured by inductively coupled mass spectroscopy (ICP-MS ELAN DRC-e, Perkin Elmer) using methane for reduction of polyatomic interferences. Calibration standards were prepared by dilution of 10 mg/l Multi-Element Calibration Standard 3 (PerkinElmer Pure Plus, PerkinElmer Life and Analytical Sciences, USA) with 1% Suprapur® HNO_3_ (Merck, Germany). Calibration curves were created using five different concentrations: 1 μg/l, 10 μg/l, 20 μg/l, 50 μg/l and 100 μg/l. Germanium (PerkinElmer Pure, PerkinElmer Life and Analytical Sciences, USA) was used as an internal standard and ClinChek® Plasma Control Level I (Recipe, Germany) was used as a reference material. Before analysis all samples were centrifuged (6000 rpm, 15 min) and the supernatant was diluted 20 times with 1% Suprapur® HNO_3_ (Merck, Germany). Technical details, optimal plasma operating and mass-spectrometer acquisition parameters are available on request.

### Statistical analysis

Differences between cases and controls were compared using the χ^2^ test or t-test, as appropriate. We created four categories of serum selenium levels based on the distribution of the controls (≤70, < 70–80, ≥80–90, > 90 μg/L). Given that we had no preconceived notion of the normal or optimal level of selenium, we selected the highest category (> 80–90 μg/L) as the reference level. Unconditional logistic regression was used to estimate the odds ratio (OR) and 95% confidence interval (CI) for any cancer according to selenium category. We also performed a secondary analysis limiting the cases to breast cancer only. Analyses were performed using SAS version 9.2 (SAS Institute, Cary, NC, USA) and all *P-*values are two-sided.

## Results

The cohort of 19,573 eligible women accumulated a total of 57,036 person-years from the date of blood draw until December 31, 2014. For the current nested case-control study, we included 97 women with a cancer diagnosis (i.e., cases) and 184 unaffected women (i.e., controls). The mean age of the female subjects at the time of blood draw was 48.6 years (range 25 to 93 years)(Table 1). Of a total of 97 incident cases (all sites) in the cohort (Table 2), 53 were new cases of breast cancer. Based on Polish age-specific incidence rates, there were 69 expected cases of breast cancer. Cases and controls were similar with respect to age at blood draw, family history of cancer, smoking status and BMI. On average 17.9 months had elapsed from the date of blood draw to the date of diagnosis in the cases (range 1.3 to 41.0 months).

**Table 1:** Selected characteristics of cases and controls

Variables	Case (*n* = 97)	Control (*n* = 184)	*P*
Data blood draw, year (range)	2011.8 (2009–2014)	2012.3 (2010–2014)	< 0.0001
Date of cancer diagnosis, year (range)	2013.3(2010.4–2014.8)	n/a	n/a
Time from blood draw to diagnosis, months (range)	17.91 (1.28–41.03)	n/a	
Cancer site, n (%)			
Breast cancer	53 (54.6%)	0	
Ovarian cancer	4 (4.1%)	0	
Other cancer	40 (41.2%)	0	n/a
Cancer in first degree relatives, n (%)			
0	35 (36.1%)	56 (30.4%)	
1	37 (38.1%)	76 (41.3%)	
2	20 (20.6%)	42 (22.8%)	
> =3	5 (5.2%)	10 (5.4%)	0.82
Breast cancer in first degree relatives, n (%)			
0	84 (86.6%)	153 (83.2%)	
1	13 (13.4%)	30 (16.3%)	
2	0	1 (0.5%)	
> =3	0	0	0.62
Smoking (current or past), n (%)			
No	53 (54.6%)	105 (56.0%)	
Yes	44 (43.4%)	87 (44.0%)	0.83

**Table 2:** Distribution of cancer sites in cases with a diagnosis of cancer during the follow-up period

Site of cancer	Number of cancers
Breast	53
Colon	9
Lung	8
Uterus	4
Ovary	4
Bladder	4
Lymphoma	3
Pancreas	2
Kidney	2
Stomach	2
Thyroid	2
Cervix	1
Skin	1
Liver	1
Gallbladder	1

**Fig. 1:**
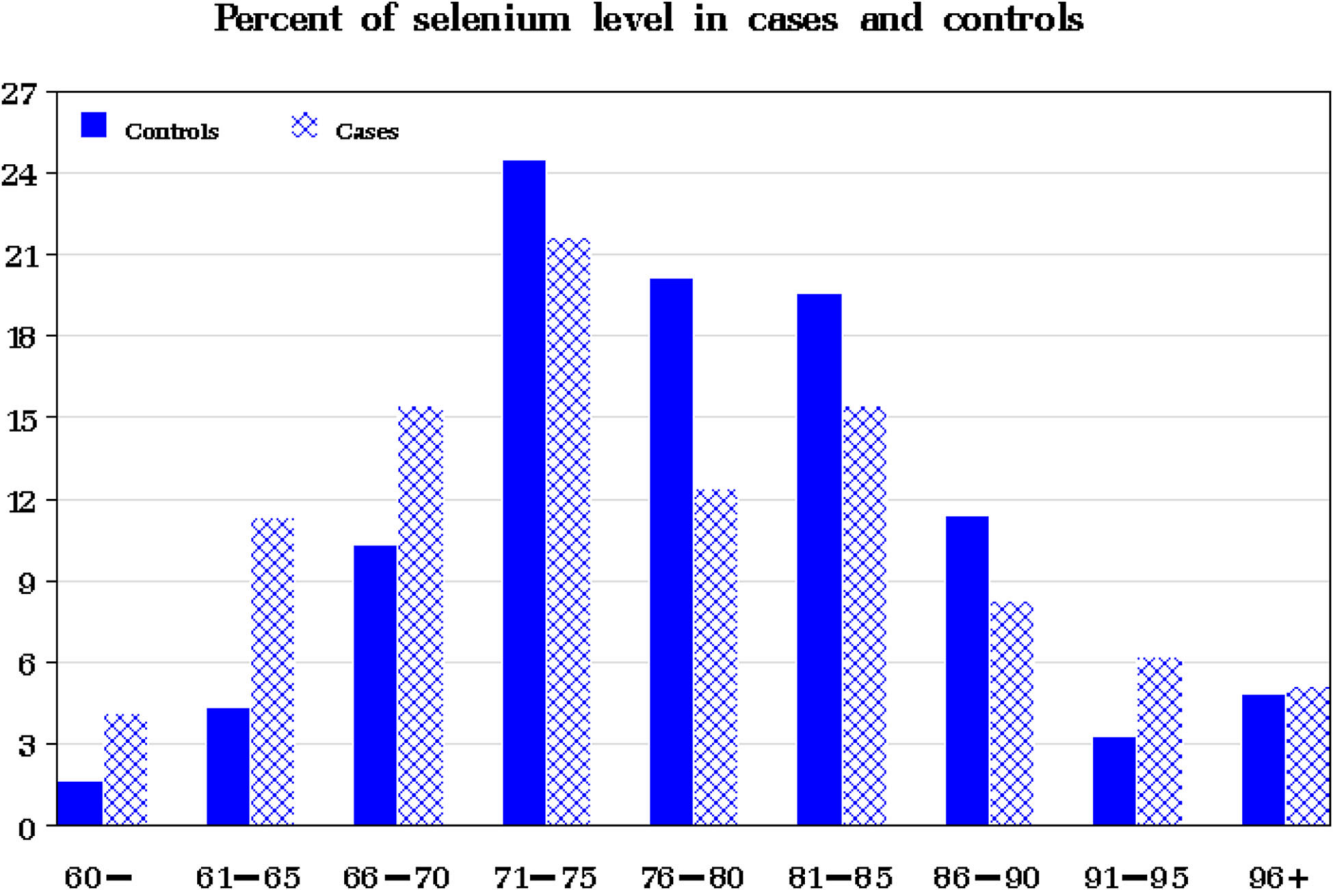
Distribution of serum selenium levels in cases and controls

The distribution of the selenium levels in the 97 cases and 184 controls is presented in Fig. 1. The mean selenium level in the cases was slightly lower than that of the controls (76.5 versus 78.3 μg/L); however, the difference was not statistically significant (*P* = 0.19).

Selenium was categorized into four categories based on the distribution in the control subjects. A case was much more likely to be in the lowest category of selenium (≤70 μg/L) than a control (30.9% versus 16.3%)(Table 3). The relationship between selenium status and cancer risk was not linear. Women in the lowest category of serum selenium levels (i.e., ≤70 μg/L) had a more than two-fold increased risk of cancer compared to those in the reference category (80–90 μg/L)(OR = 2.60; 95% CI 1.26–5.35; *P* = 0.01). The odds ratio associated being below the cutoff of 70.0 μg/L compared to a level above 70.0 μg/L was 2.29 (95% CI 1.26–4.19; *P* = 0.007). The risks for women in the two middle categories were similar and suggests that the normal range be between 70 μg/L and 90 μg/L. There was evidence for an increased risk of cancer among women in the highest category of selenium levels (i.e., > 90 μg/L), but this association did not achieve statistical significance (OR = 1.63; 95%CI 0.63–4.19; *P* = 0.31).

**Table 3:** Odds ratio for incident cancer by quartile of selenium

Selenium level (μg/L)	Cases n (%)	Controls n (%)	Univariate OR (95%CI)	*P*	Multivariate^a^ OR (95%CI)	*P*
All cancers						
≤ 70	30 (30.9%)	30 (16.3%)	2.48 (1.23–4.99)	0.01	2.60 (1.26–5.35)	0.01
70.01–80	33 (34.0%)	82 (44.6%)	1.00 (0.53–1.87)	0.99	1.13 (0.59–2.16)	0.72
80.01–90	23 (23.7%)	57 (31.0%)	1.00 (reference)		1.00 (reference)	
> 90	11 (11.3%)	15 (8.2%)	1.82 (0.73–4.54)	0.20	1.63 (0.63–4.19)	0.30
Breast cancer						
≤ 70	15 (28.3%)	30 (16.3%)	2.19 (0.92–5.20)	0.18	2.42 (1.00–5.89)	0.05
70.01–80	18 (34.0%)	82 (44.6%)	0.96 (0.44–2.12)	0.92	1.13 (0.50–2.55)	0.76
80.01–90	13 (24.5%)	57 (31.0%)	1.00 (reference)		1.00 (reference)	
> 90	7 (13.2%)	15 (8.2%)	2.05(0.69–6.03)	0.19	1.94 (0.64–5.88)	0.24

^a^Adjusted by date of blood draw

Findings were similar when the analysis was limited to breast cancer cases (Table 3). Women in the lowest category of selenium levels had an increased risk of developing breast cancer compared to those in the reference group (OR = 2.42; 95% CI 1.00–5.89; *P* = 0.05). Similarly, there was suggestive evidence for an increased risk of breast cancer among women with a selenium levels above 90 μg/L (OR = 1.97; 95%CI 0.64–5.88).

## Discussion

The results of this nested case-control study of 281 women residing in Szczecin, Poland suggests that low serum selenium levels (≤ 70 μg/L) are associated with a statistically significant two-fold increase in cancer risk. Findings were similar in an analysis restricted to breast cancer. Breast cancer was the most commonly diagnosed cancer in this cohort of women; however, most of these women were not at increased risk given that all the particpants had been tested for a *BRCA1* mutation and those with a mutation were excluded. Only 15% of the study subjects had a first-degree relative with breast cancer. Furthermore, the number of observed cases of breast cancer did not exceed the expected number (53 versus 69); however, it is possible that in our follow-up scheme that not all new cases of breast cancer were identified particularly if they were treated in a hospital outside of the Szczecin region.

Of interest is the potential U-shaped relationship between selenium status and cancer risk. Although not statistically significant, there was suggestive evidence for an increased risk of cancer among those women in the highest category of serum selenium levels (i.e., > 90 μg/L). A similar U-shaped relationship has previously been reported between selenium status, cancer incidence and all-cause mortality [4, 12, 16, 17]. The Nutritional Prevention of Cancer (NPC) trial included 1300 individuals with a personal history of basal or squamous cell carcinoma who lived in the southeast United States where soil selenium levels are low, compared to other regions [18]. In this study, the authors reported that supplemention with 200 μg of selenium per day (in the form of selenium yeast) for an average of 7.4 years resulted in a significant reduction in total cancers and prostate cancer, and to a lesser extent, cancers of the lung and colon, compared to the placebo group. Importantly, this protective effect was limited to individuals with lower baseline plasma selenium levels (defined as ≤105 ng/mL). Interestingly, there was evidence for a non-significant increased risk of other cancers including melanoma, bladder and breast cancer, among others, in the supplemented group. In contrast, an increased risk of prostate cancer incidence was reported in men who received selenium in the Selenium and Vitamin E Cancer Trial (SELECT), which included an intervention of 200 μg of selenium (in the form of selnomethionine), plus or minus vitamin E or placebo [13]. A recent report of the SELECT study confirmed that the selenium intervention increased the risk of high-grade prostate cancer in men with high baseline selenium status levels with no effect in men with low selenium status [19]. Although various features of the two trials may explain the conflicting findings including the type of selenium supplementation, effect modification by smoking and gender, differences in baseline selenium levels are likely to play an important role. For example, in the NPC trial, there was a significant interaction between treatment group (i.e., selenium supplementation vs. placebo) and risk based on baseline plasma selenium levels [4]. In other words, the protective effect of selenium was limited to those with a baseline level in the lowest tertile (i.e., ≤105.2 ng/mL) with an increased cancer risk among those with selenium in the highest tertile (i.e., > 121.6 ng/mL).

With respect to breast cancer, prospective studies have generally reported no association between blood or toenail selenium levels and risk [9, 20–23]. In our study, breast cancer was the most commonly diagnosed cancer, followed by cancers of the lung and colon. In an earlier report of individuals from the same region, we found a significant inverse (and linear) dose-response relationship between serum selenium and the occurrence of lung or laryngeal cancer [15]. Previously, we also showed that selenium levels were significantly lower in colorectal cancer patients compared with healthy controls [14]. Based on a review of the evidence, Rayman et al.*,* suggested that individuals with blood selenium levels of 122 μg/L should not be supplemented [2]. Given the findings of our current study as well as our two earlier studies, we suggest that invidiuals with serum selenium levels below 70 μg/L should be offered selenium supplementation and that the goal of the supplementation should be to arrive at a stable level of between 70 μg/L and 90 μg/L.

Because of this geographic variability in soil selenium levels, dieteray intake in Europe tends to be lower than in the United States [12, 24]. Using data from the National Health and Nutritional Examination Survey (NHANES) conducted in the United States, the mean serum selenium for women aged 40 or older was 134.7 μg/L which is substantially higher than the mean level of 78.3 μg/L reported for the controls in the current study [25].

There are several strengths of our study including the large number of unaffected women enrolled in the biobanking inititive, complemented by the collection of detailed individual participant information and fasting blood samples. We were able to assess serum selenium status which reflects short-term selenium exposure and is an accurate assessment than dietary intake which is heavily influenced by the selenium content in the soil [26, 27]. Predictors of selenium levels include age, supplement use and smoking status were controlled for in our statistical approach. Despite this, our study was not without various limitations including a relatively low number of incident cancers.

The results of the current nested case-control study suggests that the optimum level of serym selenium in women living in Poland should be between 70 μg/L and 90 μg/L and among women with low selenium levels, supplementation may easily achieve a selenium level within in this range. Approximately one third of Polish women have a level of selenium below 70 μg/L [28]. However, prior to supplementation it is advisable to measure serum selenium and selenium supplementation is not recommended for women with a value above 70 μg/L.

## Conclusion

The data presented here are observational and cannot be used to infer causality but suggest that further studies - including potentially a randomized trial - are rational. It is also important to determine to what extent selenium levels in a woman are stable over time and if the monitoring of levels after supplementation is recommended. The observation of an increased risk among women with high selenium levels is intriguing and suggests that caution need be applied to keep the selenium level between 70 and 90 μg/L. It is important that these observations be expanded in Poland and replicated in other cohorts. Our on-going follow-up of this population of women will help confirm these findings and define a safe and accuratre level of supplementation for women living in Poland.

## Data Availability

The datasets used and/or analyzed during the current study are available from the corresponding author on reasonable request.
